# Cerebrospinal Fluid Sediments as a Novel Tool for Potential Biomarkers of Neurodegenerative Diseases

**DOI:** 10.3390/ijms27083692

**Published:** 2026-04-21

**Authors:** Raquel Alsina, Marta Riba, Marina Sartorio, Clara Romera, Berta Vilaplana, Eva Prats, Laura Molina-Porcel, Jaume del Valle, Carme Pelegrí, Jordi Vilaplana

**Affiliations:** 1 Secció de Fisiologia, Departament de Bioquímica i Fisiologia, Facultat de Farmàcia i Ciències de l’Alimentació, Universitat de Barcelona, 08028 Barcelona, Spain; ralsinaplanelles@ub.edu (R.A.); msartorio@ub.edu (M.S.); cromera@ub.edu (C.R.); jdelvalle@ub.edu (J.d.V.); carmepelegri@ub.edu (C.P.); 2 Institut de Neurociències (UBNeuro), Universitat de Barcelona, 08035 Barcelona, Spain; 3 Centros de Biomedicina en Red de Enfermedades Neurodegenerativas (CIBERNED), 28031 Madrid, Spain; 4 Departament de Reumatologia, Hospital Clínic de Barcelona, 08036 Barcelona, Spain; bvilaplana@clinic.cat; 5 Unitat de Microscòpia Electrònica i Tècniques Afins, Centres Científics i Tecnològics UB (CCiTUB), 08028 Barcelona, Spain; evaprats@ccit.ub.edu; 6 Neurological Tissue Bank, Biobank of the Hospital Clínic of Barcelona-IDIBAPS, 08036 Barcelona, Spain; lmolinap@clinic.cat; 7 Alzheimer’s Disease and Other Cognitive Disorders Unit, Neurology Service, Hospital Clínic de Barcelona, Fundació de Recerca Clínic Barcelona-Institut d’Investigacions Biomèdiques August Pi i Sunyer (FRCB-IDIBAPS), Universitat de Barcelona, 08036 Barcelona, Spain

**Keywords:** cerebrospinal fluid (CSF), biomarkers, neurodegenerative diseases, Alzheimer’s disease (AD), frontotemporal lobar degeneration (FTLD), wasteosomes, corpora amylacea (CA), psammoma bodies

## Abstract

Cerebrospinal fluid (CSF) biomarkers for neurodegenerative diseases have been extensively studied over the years. However, CSF samples are routinely centrifuged, and the resulting sediment or pellet is typically discarded to remove cellular debris and high-density particles. This standard practice raises a critical question: Could these discarded sediments harbour potential biomarkers? The aim of the present study is to demonstrate that CSF sediments contain specific brain-derived components and thus to substantiate the possible presence of biomarkers within these sediments. To this end, we analysed post-mortem CSF samples of one patient with neuropathologically confirmed Alzheimer’s disease (AD) and one patient with confirmed progressive supranuclear palsy (PSP). CSF pellets were studied using transmission and scanning electron microscopy techniques (TEM and SEM, respectively), along with compositional analysis through SEM combined with energy-dispersive X-ray spectroscopy (SEM-EDX), as well as immunofluorescence and histochemical analyses on semithin pellet sections. We observed that, among others, CSF pellets contain brain-derived structures such as wasteosomes and psammoma bodies. Furthermore, we also found disease-relevant proteins, including tau and Aβ42 in the AD sediment and tau in the PSP sediment. Although further studies are required, the study of CSF pellets could open new avenues for biomarker discovery in neurodegenerative diseases.

## 1. Introduction

Neurodegenerative diseases are a heterogeneous group of neurological disorders characterised by the progressive loss of neurons in the central or peripheral nervous system. The loss of neurons and the consequent collapse of the structure and function of neural networks result in the breakdown of the core communicative circuitry, culminating in impaired memory, cognition, behaviour, sensory, and/or motor function [1]. Altogether, neurodegenerative diseases affect the lives of millions of people worldwide, and their prevalence is expected to rise with increasing life expectancy in most countries.

Among these diseases, Alzheimer’s disease (AD) stands as the most prevalent, followed by Parkinson’s disease and frontotemporal lobar degeneration (FTLD). All of these diseases share a common hallmark, as they entail the formation of insoluble aggregates of specific proteins which are often implicated in the pathology and progression of the diseases. In the case of AD, amyloid-β (Aβ) and tau aggregates constitute the major components of Aβ plaques and neurofibrillary tangles, respectively [2]. In Parkinson’s disease, aggregates of α-synuclein are the major component of Lewy bodies present in the brains of these patients [3]. Finally, for FTLD, aggregates of hyperphosphorylated tau, transactive response DNA-binding protein 43 (TDP-43), and inclusions of fused in sarcoma (FUS) protein have been reported, corresponding to the subtypes FTLD-tau, FTLD-TDP, and FTLD-FUS, respectively [4,5].

A key aspect in addressing these diseases is the identification of reliable biomarkers, as they improve diagnostic accuracy, enable early detection, and are essential for developing and monitoring disease-modifying treatments. Ongoing research and standardisation efforts aim to integrate these biomarkers into routine clinical practice, ultimately enhancing disease management and offering patients better therapeutic opportunities [6,7,8,9].

In recent years, the development of biomarkers for neurodegeneration has rapidly advanced, including neuroimaging biomarkers and fluid-based biomarkers. Of particular importance are cerebrospinal fluid (CSF) biomarkers. The underlying principle is simple: since CSF fills both the brain ventricles and the subarachnoid space and exchanges substances with the brain and spinal cord, it can potentially reflect the pathological processes occurring in these structures. Compared to blood, CSF is less prone to peripheral contamination, which theoretically should facilitate the identification and interpretation of potential biomarkers. Additionally, the concentration of key proteins of interest is higher in CSF than in blood [10].

Although research on CSF biomarkers for AD is well advanced, the availability of reliable biomarkers for other neurodegenerative disorders remains limited. This is particularly true for FTLD, where detecting the water-insoluble TDP-43 and FUS proteins in CSF presents a major challenge [4]. In this context, advances in precise analytical techniques, such as single-molecule array (Simoa) technology and seed amplification assays, which enable the detection of extremely low levels of specific components in the CSF, can be particularly useful [10,11,12,13]. These methods will also enable the detailed characterisation of these components, including aspects such as isotypes or post-translational modifications, which are particularly valuable in the context of protein aggregation [14].

Given this context, a critical fact deserves special attention and is highly relevant to our point of view. In standard CSF studies, samples are routinely centrifuged, and the sediments are discarded to eliminate cellular debris and high-density particles [14,15,16]. Thus, the question proposed is as follows: Could these discarded sediments harbour potential biomarkers relevant for the diagnosis and prognosis of certain brain diseases?

The aim of this study is to demonstrate that CSF sediments contain specific brain-derived components, thus providing a rationale for investigating potential biomarkers within these sediments.

## 2. Results

### 2.1. Representative Brain Features of the AD and Progressive Supranuclear Palsy (PSP) Donors

Based on the neuropathological diagnosis, we first performed indirect immunostaining for Aβ and tau on specific hippocampal sections from both donors. This allowed us to visualise the representative brain features of each case in terms of tau and Aβ deposits and to validate the antibodies that would later be used for staining the CSF pellets from both donors. For the Aβ staining, we used the 6E10 antibody, directed against the different isoforms of Aβ, and the 12F4 antibody, specifically targeting the Aβ42 isoform. For tau staining, we used the Tau5 antibody, directed against the tau protein. Preadsorption procedures were conducted to confirm the specificity of these stains, and negative controls were performed by incubating the samples with the buffer solution instead of the primary antibody.

All of these stains were combined with that of ubiquitin-binding protein p62 (p62), which is a well-established marker of brain wasteosomes [17,18]. Wasteosomes, originally known as corpora amylacea, are bodies that accumulate in the human brain and can be thereafter released into the CSF [17,19]. Therefore, it is expected that they can be detected both in the hippocampal sections and in the CSF pellets that will subsequently be analysed.

**Figure 1:**
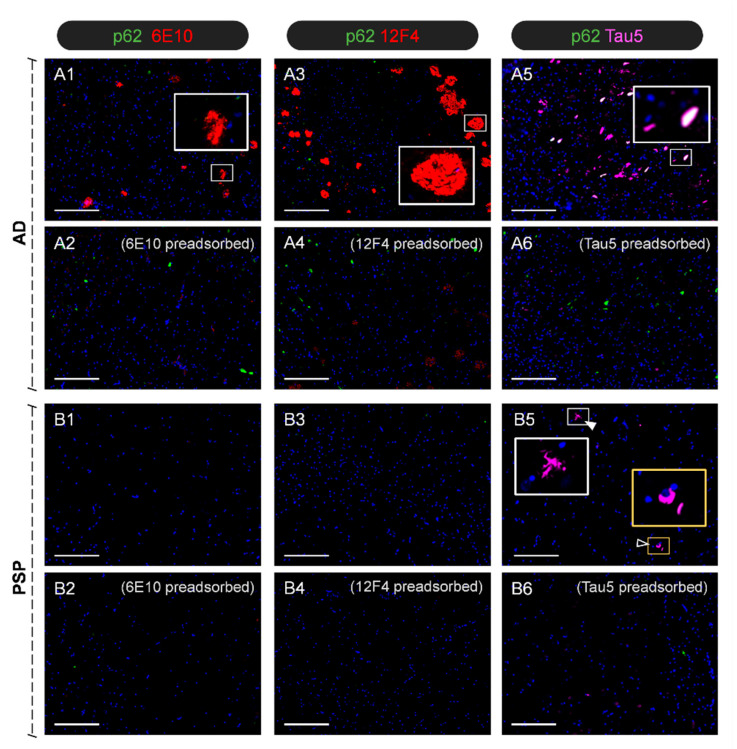
Representative images of immunofluorescence staining of the CA1 region of hippocampal sections. In some images, some insets are magnified to facilitate the visualisation of specific structures. The AD patient showed the characteristic Aβ plaques stained with 6E10 and 12F4 (**A1**,**A3**), whereas the PSP patient did not display these plaques (**B1**,**B3**). Preadsorption of both antibodies with the Aβ42 peptide (**A2**,**A4**,**B2**,**B4**) abolished the staining observed in the AD case. Regarding tau, the AD case exhibited abundant and widespread NFTs, some of which can be observed in white colour because they also become stained with α-p62 (**A5**). Staining with Tau5 in the PSP case showed the characteristic tufted tau-positive astrocytes and globose-shaped NFTs (**B5**) (arrowhead and empty arrowhead, respectively). Tau staining disappeared in both AD and PSP cases after preadsorption of Tau5 with the tau protein (**A6**,**B6**). All sections contain Hoechst staining (blue). Scale bars: 200 µm.

As expected, and illustrated in Figure 1, the hippocampus of the AD patient contains Aβ plaques that become visible with 6E10 and 12F4 staining, and it also contains the characteristic neurofibrillary tangles (NFTs), which become visible with Tau5 staining. The α-p62 antibody stains some of the NFTs present in the AD hippocampus, and thus some NFTs become double-stained with both the α-p62 and Tau5 antibodies. In the PSP sections, there are no 6E10 and 12F4 stains because of the absence of Aβ plaques in the hippocampus, while Tau5 staining permits the observation of both the characteristic globose NFTs and tau-positive tufted astrocytes. In all cases, the preadsorption of the primary antibodies with the respective targeted protein abolished the staining.

### 2.2. A First Inspection of the CSF Pellets

For a preliminary inspection of the CSF pellets, we stained some semithin sections from each pellet with the periodic acid–Schiff (PAS) technique. It should be noted that, in the present study, PAS staining was performed on semithin sections; therefore, the staining intensity is reduced compared to that observed in standard histological sections.

**Figure 2:**
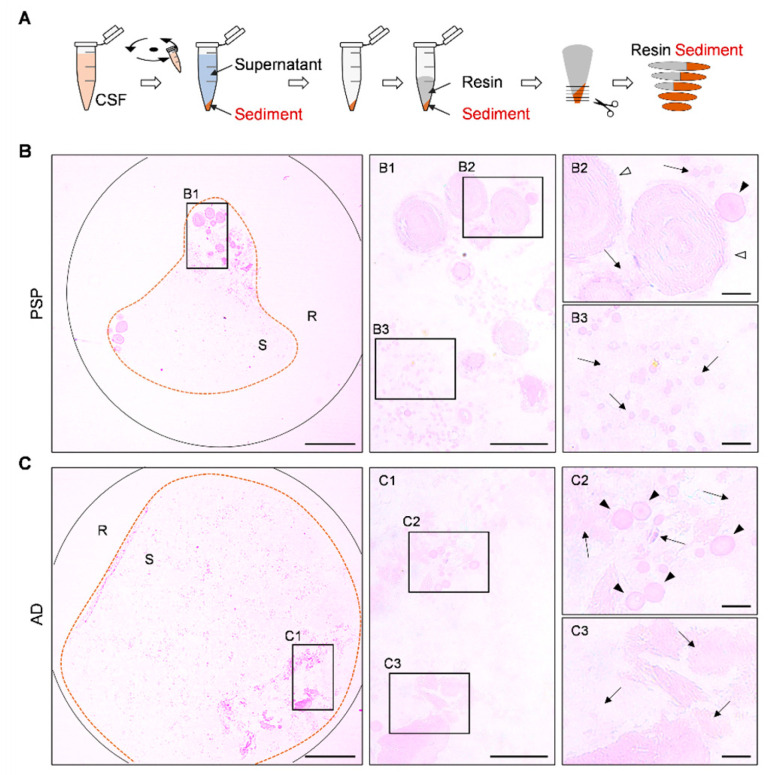
Representative images of semithin sections obtained from CSF pellets and stained with PAS: (**A**) To obtain the semithin sections, CSF samples are centrifuged, the supernatant is then eliminated, and the pellet is resin-embedded to obtain the sections (500 nm thick). (**B**,**C**) Representative images obtained from the PSP and AD donors. These circular sections include a sediment area (S), as well as a region containing only resin (R). As can be observed in the insets (**B1**–**B3**,**C1**–**C3**), the sediments contain wasteosomes (arrowheads) and psammoma bodies (empty arrowheads), alongside other unidentified pellet deposits (arrows). Scale bars: 400 µm on (**B**,**C**); 100 µm on (**B1**,**C1**); 50 µm on (**B2**,**B3**,**C2**,**C3**).

As illustrated in Figure 2, the staining of both the AD and PSP samples revealed scattered, lightly stained components alongside structures with more intense staining. Among these structures, wasteosomes were identified, some of them within the pellet deposits and others at the edges of the semithin sections, likely due to redistribution during the centrifugation step of sample preparation. Additionally, in the pellets from both patients, as well as in additional cases not included in this study, PAS staining revealed faintly stained structures consistent with psammoma bodies. It should be noted that the presence of wasteosomes in the CSF and their brain origin had already been documented [17]; however, the presence of psammoma bodies in CSF pellets, which was confirmed with our subsequent tests, had not been previously described.

### 2.3. Ultrastructural Characterisation of CSF Pellets Using Transmission Electron Microscopy (TEM)

Based on the observations made using the PAS staining technique, certain regions of interest (ROIs) have been identified in the different semithin sections, and ultrathin sections of these ROIs have been obtained and processed for visualisation under TEM.

**Figure 3:**
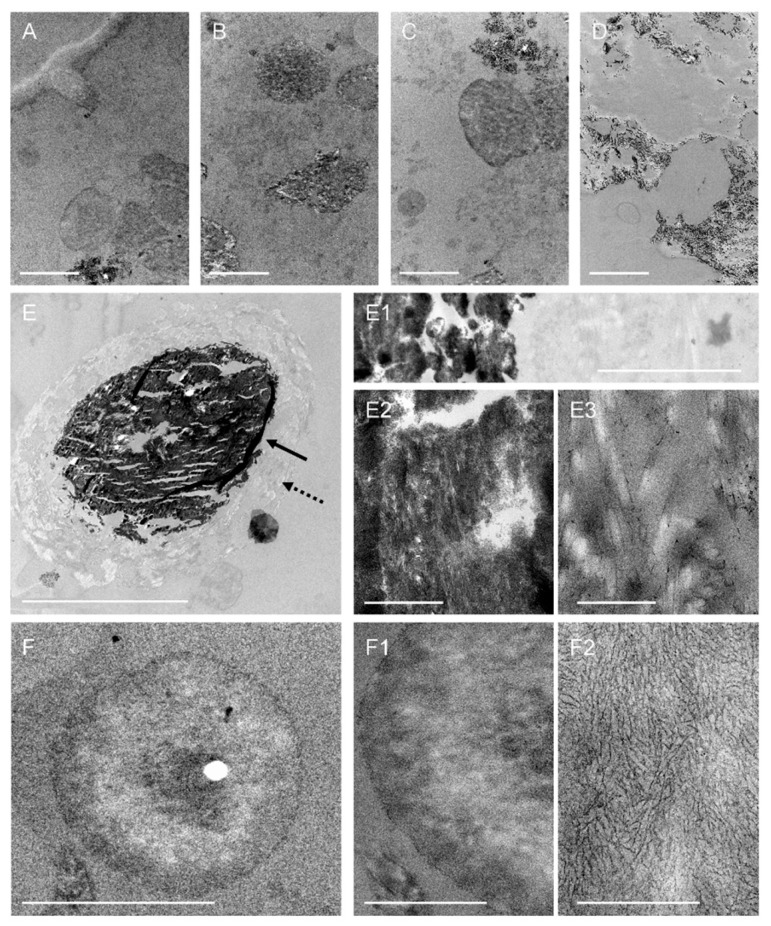
Representative TEM images of the CSF pellet obtained from the PSP case: (**A**–**D**) TEM images highlighting amorphous structures and various remnants in different regions of the sample. Scale bars: 5 µm. (**E**) Among the different structures, some are compatible with psammoma bodies. In this case, the psammoma contains a central electrodense region (arrow) and a peripheral non-electrodense region (dashed arrow). Scale bar: 20 µm. (**E1**) Higher-magnification TEM image that includes the electrodense region of the psammoma body and its peripheral region. Scale bar: 2 µm. (**E2**,**E3**) At higher magnification, fibrillar structures appear in both the central and the peripheral regions of the psammoma. Scale bars: 500 nm. (**F**) Representative TEM image showing a wasteosome. Scale bar: 20 µm. (**F1**,**F2**) Higher-magnification TEM images showing the detailed ultrastructure of the wasteosome. As can be observed in (**F2**), wasteosomes encompass densely packed, randomly oriented, short linear fibres. Scale bars: 20 µm (**F1**) or 500 nm (**F2**).

As illustrated in Figure 3 for the PSP case, the TEM analysis of the ultrathin sections of the CSF pellets from both donors revealed not only a variety of amorphous structures but also membranous debris, fibrillar structures, and vesicles, as well as wasteosomes and psammoma bodies.

**Figure 4:**
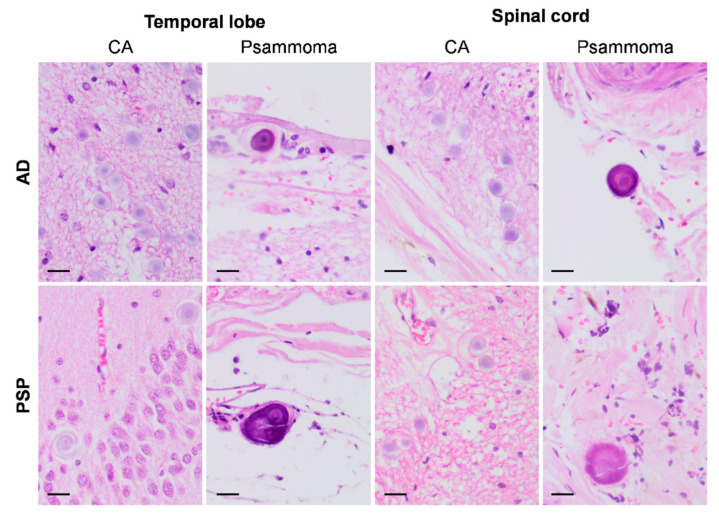
Representative images from haematoxylin-and-eosin-stained sections from the temporal lobe and the spinal cord. The first row displays images from the AD patient, with the first two columns featuring a wasteosome or corpora amylacea (CA) and a psammoma body in the temporal lobe, and the third and fourth columns showing the same features in the spinal cord. The second row shows similar images from the PSP patient. Scale bar: 20 µm.

To investigate further, we analysed haematoxylin-and-eosin-stained sections from the temporal lobe and spinal cord of the studied donors (sections previously used in the donors’ neuropathological examinations) and observed the presence of both wasteosomes and psammoma bodies in both structures, located either within the parenchyma or along the bordering regions (Figure 4). These observations support the brain origin of the psammoma bodies found in the CSF and strongly reinforce the presence of brain-derived components in the CSF pellets.

### 2.4. Characterisation of CSF Pellets Using Scanning Electron Microscopy (SEM) Combined with Energy-Dispersive X-Ray Spectroscopy (SEM-EDX)

**Figure 5:**
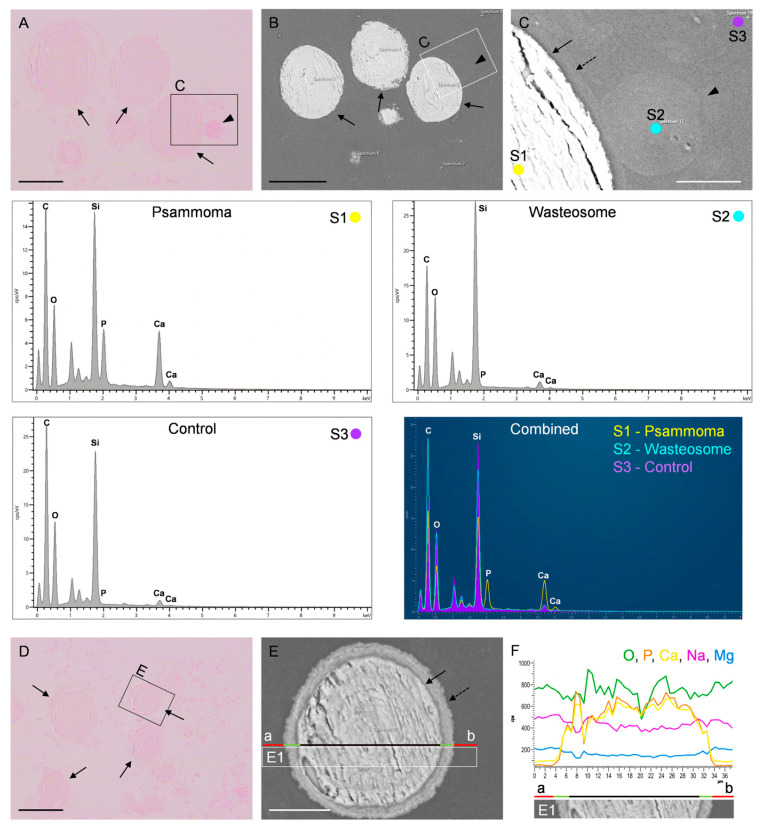
Representative images of SEM and SEM-EDX of the CSF pellet from the PSP case: (**A**) PAS-stained semithin section in which a wasteosome and some psammoma bodies can be observed (arrowhead and arrows, respectively). (**B**) SEM image of the equivalent region, obtained in a consecutive section. (**C**) SEM image corresponding to the C area marked in (**A**,**B**). The dashed arrow indicates the peripheral region of a psammoma, the black arrow its core, and the arrowhead indicates a wasteosome. The SEM-EDX spectra obtained in the S1, S2, and S3 points (corresponding, respectively, to the psammoma, wasteosome, and control regions) are shown below. Note the high levels of P and Ca in the psammoma body, clearly visible on the combined graph. (**D**) PAS-stained semithin section showing various psammoma bodies (black arrows). The psammoma body included in the area labelled as (**E**) was further analysed by SEM-EDX. (**E**) SEM image of this psammoma body, obtained in a consecutive slide. The dashed arrow indicates its peripheral region, and the black arrow indicates its core. (**F**) Profile of the SEM-EDX analysis along the a–b line indicated in (**E**). Note that, in the core of the psammoma (line in black), the levels of P and Ca are increased when compared with those of the control region (line in red). The levels of such components show intermediate values in the peripheral region of the psammoma (line in green). E1 corresponds to the inset signalled in (**E**). Scale bars: 50 µm (in black) or 10 µm (in white).

Following TEM analysis, complementary observations were made using SEM imaging and SEM-EDX analysis. As expected, the SEM images permit the identification of the presence of wasteosomes and psammoma bodies along with some unidentified remnants in the pellets from both the AD and the PSP patients. Figure 5 illustrates the results of these studies for the PSP case and shows the presence of both types of structures (psammoma bodies and wasteosomes) in representative sections stained with PAS, the magnifications of some of these structures in consecutive sections observed on SEM, and some of the spectra obtained with the analysis performed by SEM-EDX.

In the SEM-EDX analysis, we observed that the spectrum of wasteosomes does not differ from that of the surrounding region (control region). It is important to note that the main components of wasteosomes are carbohydrates; therefore, a high presence of carbon (C), hydrogen (H), and oxygen (O) would be expected. Nonetheless, the detection of carbon is masked by the carbon coating required for analysing non-conductive samples. In the case of O, it can be seen that the control region shows a high presence of silicon (Si) and O due to the glass slide, and this O masks the signal from the O present in the wasteosomes. On the other hand, hydrogen, due to its low molecular weight, cannot be reliably detected by SEM-EDX. In contrast, the SEM-EDX analysis did reveal significant differences between psammoma bodies and the control regions. As shown in Figure 5, there is a high presence of calcium (Ca) and phosphorus (P) in the core of the psammoma bodies, supporting their identification as such.

### 2.5. Immunofluorescence on CSF Pellets

After confirming the presence of both wasteosomes and psammoma bodies in the CSF, we studied the presence of specific components using immunofluorescence on CSF semithin sections.

**Figure 6:**
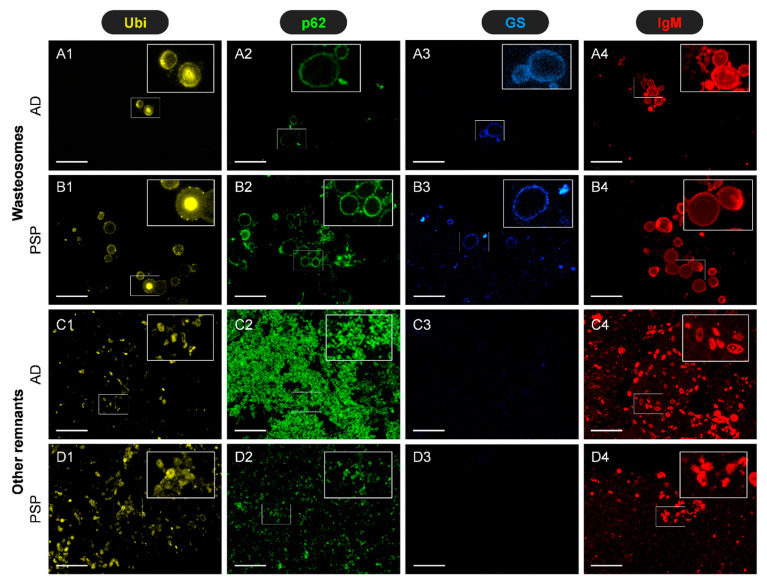
Representative images of immunostained semithin sections of CSF pellets from AD and PSP donors: (**A1**–**A4**) Images from the AD donor showing the staining of wasteosomes with α-Ubi, α-p62, α-GS, and IgMs, respectively. One region of each image (inset) is magnified and the colour adjusted to facilitate its visualisation. (**B1**–**B4**) Images from the PSP donor showing the stainings of wasteosomes with α-Ubi, α-p62, α-GS, and IgMs, respectively. One region of each image (inset) is magnified, and the colour is adjusted to facilitate its visualisation. (**C1**–**C4**,**D1**–**D4**) Illustration of the presence of staining in other regions of the pellet in the AD and PSP sections, respectively. Note the staining of wasteosomes in the sections from both donors and the presence of some remnants that become stained by α-Ubi, α-p62, and IgM. Scale bar: 50 µm.

To begin with, we performed specific immunostainings based on previous studies. Specifically, we previously observed that after resuspending the pellet and spreading it onto a slide, wasteosomes present in CSF can be immunostained with antibodies α-ubiquitin (α-Ubi), α-p62, α-glycogen synthase (α-GS), and natural IgMs [17]. Therefore, as a first step, we tested whether these immunostainings could also be observed in wasteosomes sectioned in the semithin slices used for our analyses. As illustrated in Figure 6, the results indicate that wasteosomes on the semithin sections from both donors indeed exhibit well-defined positive labelling with α-Ubi, α-p62, and IgMs. In contrast, GS staining was very weak. As expected, staining with α-Ubi, α-p62, and IgMs appears in the peripheral regions of wasteosomes, showing well-defined circular or ring-like structures with uniform staining. Moreover, some wasteosomes present a central core that becomes stained with Ubi but not with the other antibodies. The staining of GS, although faint, is also located in the peripheral regions of wasteosomes. Altogether, these observations validate the use of immunofluorescence techniques on these semithin sections. Moreover, it is important to highlight that, in addition to the labelling observed in wasteosomes, there is also some labelling in other regions of the pellets (also illustrated in Figure 6). Thus, staining with IgMs reveals numerous circular deposits scattered across the field, often accompanied by smaller, punctate, and less organised structures. Ubi staining exhibits a similar pattern to that observed with natural IgMs. Likewise, p62 staining displays diffuse granular patterns. Notably, no GS staining is observed in the pellet deposits. The presence of such components that become stained with α-Ubi, α-p62, and IgMs is consistent with the presence of remnants in the pellet, as all of these stains are related to the signalling and removal of waste products [17,18,20].

### 2.6. Presence of Disease-Related Proteins in CSF Pellets

Given the remnants observed in both the ultrathin and semithin sections of the CSF pellets with different techniques, and the possibility that they contain information overlooked by the standard techniques commonly used, we further studied the presence of some disease-related proteins in these remnants. As is well known, both Aβ and tau proteins are affected in AD, while only the latter is affected in PSP. Accordingly, we performed 6E10, 12F4, and Tau5 immunostaining on semithin sections, adding both preadsorption experiments to verify the specificity of the stainings and the respective controls by incubating the samples with a blocking buffer (BB) instead of the primary antibodies. As mentioned earlier, 6E10 and 12F4 antibodies are directed against Aβ and the isoform Aβ42, respectively, while Tau5 is directed against the tau protein.

**Figure 7:**
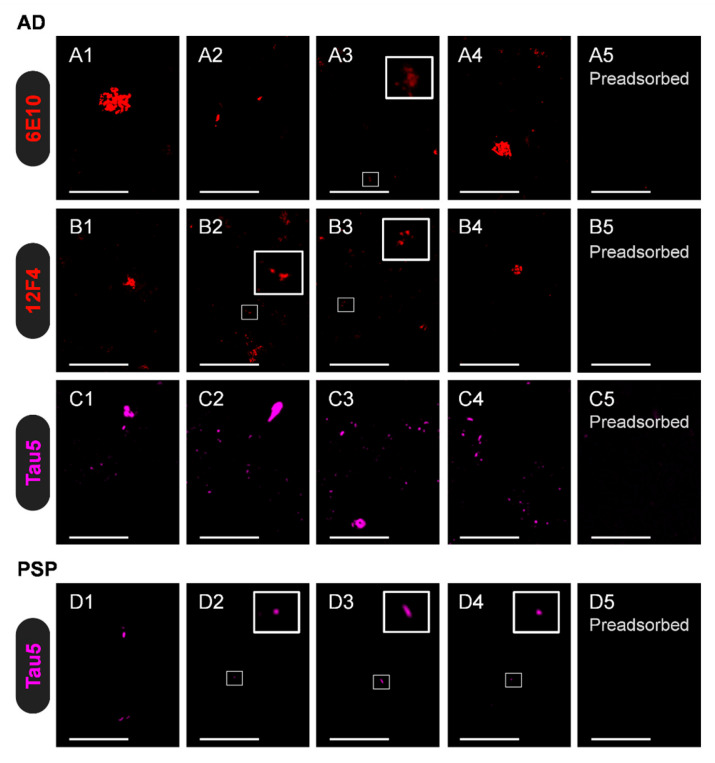
Representative images of immunostained semithin sections of CSF pellets from the AD and PSP donors. Note that in some images, some insets are magnified and the colour adjusted to facilitate the visualisation of specific components that otherwise remain unappreciated: (**A1**–**A4**) Sections from the AD donor immunostained with 6E10 show varied positive structures. (**A5**) When preadsorbing 6E10 with Aβ42, there is no staining. (**B1**–**B5**) The same procedure, but with the 12F4 antibody, produces similar results: some components become immunostained, and the preadsorption of the 12F4 antibody leads to the absence of staining. (**C1**–**C5**) When using Tau5, some tau-positive structures can be observed, and the preadsorption with the tau protein prevents such staining. (**D1**–**D5**) For the PSP donor, staining with Tau5 also leads to the staining of some structures, which mainly disappear when the antibody is preadsorbed with the tau protein. The 6E10 and 12F4 antibodies do not lead to any staining on the PSP sections. Altogether, these results suggest that CSF pellets from AD contain both Aβ and tau proteins, whereas those from PSP contain only tau protein, closely reflecting the characteristic protein profiles of each disease. Scale bar: 50 µm.

Notably, immunostaining with 6E10 and 12F4 revealed that the semithin sections from the AD patient, but not those from the PSP patient, contained irregular and amorphous structures labelled by both antibodies. As these stainings disappear when the primary antibodies are preadsorbed with Aβ42, these findings indicate the presence of Aβ42 in the pellets of the AD donor. On the other hand, immunostaining with Tau5 revealed the presence of tau in the pellets from both the AD and PSP donors, with particularly strong labelling in the AD donor. During the preadsorption studies with the tau protein, the disappearance of tau labelling was evident in both the AD and PSP pellets, although some background or nonspecific labelling was noted in the PSP pellet. The positive stainings and preadsorption results are summarised in Figure 7. Altogether, these results indicate that the CSF pellet from AD contains both Aβ and tau proteins, whereas that from PSP contains tau protein. Accordingly, the results suggest that pellets could contain the characteristic protein profiles of each disease, although further studies are required.

## 3. Discussion

The findings of the present work indicate that CSF pellets can contain a wide variety of components, including membranous debris, fibrillar structures, and vesicles, as well as wasteosomes and psammoma bodies, and they may also contain some disease-specific proteins. Specifically, the CSF pellet from the AD case contains Aβ, particularly Aβ42, which has very low solubility and tends to aggregate. Additionally, tau protein may be present in CSF pellets, as evidenced by both the strong tau labelling observed in the semithin sections from the AD donor and the faint staining observed in the PSP donor. These findings demonstrate that CSF sediments contain specific brain-derived components. Accordingly, although further studies are required, the study of CSF pellets may open new avenues for biomarker discovery in neurodegenerative diseases.

At this point, it should be noted that the CSF samples used in the study are intraventricular fluid samples obtained postmortem. The use of postmortem samples allowed us to analyse brain tissue and CSF from the same patients while also providing a confirmed neuropathological diagnosis. Future studies need to be directed towards the presence of these components in the pellets of CSF samples obtained by lumbar puncture in living people. Notably, it should be mentioned that material exhibiting some characteristics of Aβ had previously been noted in sediments of CSF samples obtained by lumbar puncture, based on observations made in electron microscopy and staining of the sediment with Thioflavin S [21]. Furthermore, in studies that we are carrying out, we have observed that CSF samples obtained by lumbar puncture contain wasteosomes, and therefore we know that their solid fraction or sediment contains components of cerebral origin.

Moreover, regarding Aβ in the CSF, it is widely accepted that AD patients exhibit a reduction in Aβ42 in the supernatant fraction of the CSF, with the prevailing hypothesis being that Aβ aggregation and retention in plaques in the brain parenchyma reduce its diffusion into the CSF [22]. Our results suggest a complementary explanation: part of Aβ is sequestered in the sediment following CSF centrifugation. This process may be facilitated by the “seeded polymerisation”, which transforms soluble Aβ monomers into insoluble monomers and fibrillar aggregates, taking place not only in the brain parenchyma but also in the CSF itself [23,24]. Additionally, because these aggregates in the CSF must be relatively large, they are likely to be cleared through the meningeal lymphatics and phagocytosed by meningeal macrophages or transported to the cervical lymph nodes and beyond [25,26,27,28]. Notably, a recent study indicates that fluid biomarkers are enriched in human cervical lymph nodes, with tau levels in lymph fluid being 266 times higher than in blood [29]. This finding aligns with our observations, suggesting that tau may be present in the CSF at higher concentrations than clinically detected, as a fraction accumulates in the sediment. Thus, although further studies are needed to determine the potential advantages of measuring these or other proteins or components in CSF pellets, the results obtained justify further investigation in this area.

In addition to disease-related proteins, our results indicate that CSF pellets may also contain wasteosomes and psammoma bodies. The presence of wasteosomes in CSF pellets from patients with AD has already been reported [17]. Wasteosomes are brain structures whose quantity increases with age and is further elevated in the context of various neurodegenerative diseases [30,31,32,33,34,35,36,37,38,39]. Proposed to be a hallmark of chronic glymphatic insufficiency [40], they act as waste containers and can serve to remove waste products from the brain [17]. In fact, they can be released from the brain into the CSF [17,19]; from there, they can be transported to cervical lymph nodes via the meningeal lymphatic system [17]. Additionally, they have been shown to undergo phagocytosis by macrophages in vitro [17,41,42,43].

Consistent with their proposed role as waste containers, several studies have shown that wasteosomes from the brains of individuals with certain diseases can contain proteins associated with those diseases. For example, in the brain of patients with AD, wasteosomes may contain tau protein, although not Aβ [36,39]; in cases of FTLD, they may harbour traces of tau, FUS, or TDP-43, depending on the FTLD subtype [31]. Based on these findings, it would be reasonable to expect that some of the wasteosomes observed in the CSF pellets might contain tau protein in AD cases and possibly in PSP cases as well. However, in the sections we analysed, we were unable to detect tau within the wasteosomes. It should be noted that the presence of tau in the wasteosomes found in the brains of disease-affected donors was not a general feature; only a few of them contained the protein. Therefore, finding wasteosomes containing this protein in the CSF would be unlikely.

In reference to psammoma bodies, their presence in CSF pellets has not been previously reported. These structures are commonly found in both neoplastic and non-neoplastic conditions and have been particularly associated with meningiomas, age-related changes, and epithelial atrophy [44,45,46,47]. In the medulla oblongata region, they appear in the nipple of the choroid plexus on the roof of the fourth ventricle [48]. Given that the choroid plexus serves as the primary site of CSF production and secretion, the detection of psammoma bodies in CSF pellets is not unexpected. Moreover, the presence of psammoma bodies is not only restricted to this choroid plexus-related region. As shown in the Section 2, we also observed them in the temporal lobe and spinal cord, located either within their parenchyma or in their bordering regions; we do not rule out their presence in other unexamined brain regions. This evidence also reinforces the brain origin of the psammoma bodies observed in the CSF pellets.

## 4. Materials and Methods

### 4.1. Experimental Design

To investigate whether CSF sediments contain brain-derived components, CSF samples from different donors were examined. The study involved ultrastructural characterisation of CSF pellets using TEM and SEM, along with compositional analysis through SEM-EDX. Additionally, semithin sections (500 nm) were prepared and processed using an adapted electron microscopy protocol to enable chemical and immunofluorescence staining, allowing for a detailed examination of the molecular and structural components present in the CSF sediments. In addition to the analysis of CSF pellets, brain samples of the corresponding cases were also used to reinforce and contextualise the findings observed in the CSF samples.

### 4.2. Human Brain Samples

Postmortem cryopreserved hippocampal sections (6 µm thick) were obtained from one neuropathologically confirmed case of AD with Down syndrome (female, 64 years old; 24:00 hh/mm postmortem delay) and one from FTLD-tau, specifically with PSP (female, 88 years old; 05:14 hh/mm postmortem delay). The diagnosis and disease progression of both donors were confirmed postmortem through neuropathological examination. The neuropathological assessment followed standardised protocols established by the Neurological Tissue Bank (Biobank-Hospital Clínic-IDIBAPS, Barcelona, Spain) as well as international consensus criteria [49]. Briefly, half of each donor’s brain was fixed in a 4% formaldehyde solution for three weeks, after which paraffin-embedded sections (5 µm thick) were prepared from at least 25 brain regions to support the diagnosis. The other half of the brain was freshly dissected. A fragment of the hippocampus was collected and immersed in 4% paraformaldehyde (4 °C) for 24 h, followed by a 48 h immersion in 30% sucrose in phosphate-buffered saline (PBS) (4 °C). Once dried, the hippocampal fragment was frozen at −20 °C and then stored at −80 °C. Subsequent cryostat sectioning at 6 µm enabled their use in immunofluorescence and histochemical analyses. All procedures, including the preparation and storage of hippocampal samples, were conducted by the Neurological Tissue Bank (Biobank-Hospital Clínic-IDIBAPS, Barcelona, Spain).

Brain tissue and CSF samples (see below) were collected with written informed consent from the patients or their legal representatives, permitting the use of brain tissue and medical records for research, as authorised by the Ethics Committee of the Neurological Tissue Bank of IDIBAPS Biobank, following the Declaration of Helsinki. The experiments involving human tissue complied with relevant ethical guidelines and regulations and received approval from the Bioethical Committee of the University of Barcelona (IRB00003099).

### 4.3. Human CSF Samples

Postmortem intraventricular CSF samples were collected from the same donors from whom hippocampal sections were obtained. Samples were initially centrifuged at 1000× *g* for 5 min, and the supernatants were discarded. Pellets were then resuspended in 0.1 M phosphate buffer (PB) and centrifuged again at 700× *g* for 10 min, with the supernatants discarded. This washing step was repeated twice. Next, pellets were fixed in 4% paraformaldehyde in PB for 1 h with gentle agitation. Afterwards, pellets were centrifuged at 700× *g* for 10 min, and the supernatants were discarded. Pellets were stored at 4 °C and transported to the Electron Microscopy Unit (UME) at the Scientific and Technological Centres of the University of Barcelona (CCiTUB). They were then washed four times in deionised water for 10 min each, followed by dehydration in a graded ethanol series. Finally, pellets were embedded in EPON resin and polymerised in a heater. Semithin sections (500 nm thick) were prepared from each sample using an ultramicrotome (EM UC7, Leica, Wetzlar, Germany) with a digital camera (IC90 E CMOS, Leica, Wetzlar, Germany).

### 4.4. Immunofluorescence in Brain Samples

Immunofluorescence studies on brain samples include immunostaining with antibodies directed against Aβ, tau, and p62 proteins. As a first step, the hippocampal sections were air-dried for 10 min at room temperature. For Aβ staining, samples were rehydrated in PBS for 5 min, incubated in 70% formic acid for 30 s, and then briefly rinsed by immersion and washed in PBS for 5 min. For tau staining, sections were placed in a water bath set at 100 °C inside staining dishes containing citrate buffer (pH of 6.0) for 7.5 min. The staining dishes were then removed from the water bath and left at room temperature for 20 min. After cooling down, the samples were washed with PBS. After the respective pre-treatments, hippocampal sections were blocked and permeabilised with 1% bovine serum albumin (Sigma-Aldrich, Burlington, MA, USA) in PBS BB containing 0.1% Triton X-100 (Sigma-Aldrich) for 20 min. Samples were then washed with PBS and incubated for 21 h at 4 °C with the primary antibodies for double staining: a mouse monoclonal IgG1 against tau (clone Tau5; 1:200; AHB0042; Thermo Fisher Scientific, Waltham, MA, USA), a mouse monoclonal IgG1 against Aβ16 (clone 6E10; 1:500; 803001; BioLegend, San Diego, CA, USA), or a mouse monoclonal IgG1 against Aβ42 (clone 12F4; 1:100; 805501; BioLegend), combined with a mouse monoclonal IgG2a against p62 (clone 2C11; 1:250; ab56416; Abcam, Cambridge, UK). Sections were then washed and incubated for 1 h at room temperature with the corresponding secondary antibodies: AF555 goat anti-mouse IgG1 (1:250; A-21127; Life Technologies, Carlsbad, CA, USA) and AF488 goat anti-mouse IgG2a (1:250; A-21131; Life Technologies). Nuclei were then stained with the Hoechst stain (2 μg/mL; H-33258; Fluka, Madrid, Spain), and the samples were washed and coverslipped with Fluoromount (Electron Microscopy Sciences, Morgantown, PA, USA). Staining controls were performed by incubating with BB instead of the primary antibody before incubation with the secondary antibody.

Additionally, preadsorption studies were performed to verify the specificity of the staining. Specifically, 6E10 and 12F4 antibodies were incubated for 21 h at 4 °C with a 100-fold molar excess of Aβ42 peptide, then centrifuged at 16,000× *g* for 30 min before being used for immunofluorescence. The same procedure was applied to the anti-tau antibody, with a 20-fold molar excess of tau protein.

### 4.5. PAS Staining in CSF Semithin Sections

Semithin sections (500 nm thick) were de-embedded by incubating in a 3:1 mixture of sodium methoxide and toluene/methanol for 5 min, followed by rinsing with toluene/methanol for 5 min, acetone for two 5 min washes, and a final water rinse. Subsequently, sections were stained using the PAS method, following the standard procedure previously described [49]. Briefly, the sections were fixed in Carnoy’s solution (60% ethanol, 30% chloroform, and 10% glacial acetic acid) for 10 min. They were then pre-treated with 0.25% periodic acid (19324-50, Electron Microscopy Sciences) in deionised water for 10 min, followed by a 3 min wash with deionised water. The samples were then incubated in Schiff’s reagent (26052–06, Electron Microscopy Sciences) for 10 min and washed for 5 min with deionised water. Nuclei were counterstained for 1 min with Mayer’s haematoxylin solution (3870, J. T. Baker, Center Valley, PA, USA). Afterward, the samples were washed, dehydrated with xylene, and mounted with Eukitt mounting medium (03989, Merck, Darmstadt, Germany).

### 4.6. Immunofluorescence in CSF Semithin Sections

CSF semithin sections were immunostained for ubiquitin Ubi, p62, GS, neoepitopes, Aβ, and tau proteins. In all cases, as a first step, semithin sections (500 nm thick) were de-embedded by incubating in a 3:1 mixture of sodium methoxide and toluene/methanol for 5 min, followed by rinsing with toluene/methanol for 5 min, acetone for two 5 min washes, and a final water rinse. For Ubi, p62, GS, and neoepitope stainings, semithin sections were blocked and permeabilised with BB containing 0.1% Triton X-100 for 20 min. Samples were then washed with PBS and incubated for 21 h at 4 °C with the following primary antibodies: a mouse monoclonal IgG1 against Ubi (clone Ubi-1; 1:200; ab7254; Abcam), a rabbit monoclonal IgG2a α-p62 (clone EPR4844; 1:250; Ab109012; Abcam), a rabbit monoclonal IgG against GS (clone 15B1; 1:100; 3886S; Cell Signaling, Danvers, MA, USA), and a pool of human IgMs containing natural IgMs that bind to certain neoepitopes present in wasteosomes (1:25; 18260-1MG; Merck). Sections were then washed and incubated for 1 h at room temperature with the corresponding secondary antibodies: AF555 goat anti-mouse IgG1 (1:250; A-21127; Life Technologies), AF555 donkey anti-rabbit IgG (1:250; A-31572; Life Technologies), and AF594 goat anti-human IgM (1:250; A-21216; Life Technologies). Samples were then washed and coverslipped with Fluoromount (Electron Microscopy Sciences; Hatfield, PA, USA). Staining controls were performed by incubating with BB instead of the primary antibody before incubation with the secondary antibody. For Aβ and tau staining, semithin sections followed the same protocol as hippocampal sections, except for the water bath time in the tau pre-treatment, which was 20 min.

### 4.7. Image Acquisition

Immunohistochemistry and fluorescence images were captured using a fluorescence laser and optical microscope (BX41, Olympus, Hamburg, Germany) and stored in .tiff format. Exposure time was adjusted for each specific stain, with corresponding control images acquired using the same exposure settings. Image processing and analysis were conducted using ImageJ software (version 1.54p, National Institutes of Health, Bethesda, MD, USA) [50]. Any adjustments to contrast and brightness for improved visualisation were applied uniformly to both the experimental and control images.

### 4.8. TEM Procedures

The semithin sections were first embedded in a Spurr resin block, which was subsequently trimmed to a 200 µm^2^ area. Ultrathin sections (60 nm) were then obtained using an ultramicrotome (EM UC7, Leica, Wetzlar, Germany) with a digital camera (IC90 E CMOS, Leica, Wetzlar, Germany). The ultrathin sections were mounted on a 200-mesh copper grid and a 200-mesh slot copper grid. Finally, they were stained with uranyl acetate for 4 min, followed by lead citrate for 2 min, and then washed. The TEM analysis was conducted using a transmission electron microscope (JEM 1010 80 kV, JEOL, Tokyo, Japan) equipped with a side-mounted camera (Orius SC200D CCD, Gatan, Pleasanton, CA, USA). Imaging was performed at an acceleration voltage of 80 kV.

### 4.9. SEM-EDX Procedures

The semithin sections were first coated with a carbon thin film to improve their electrical conductivity using a carbon evaporator (Q150Tplus, Quorum, Lewes, UK). Then, the sections were mounted on the holder using a double-stick carbon tape and placed into the chamber. The SEM analysis was conducted using a Schottky-type field SEM (JSM-7100F, JEOL, Tokyo, Japan) equipped with an EDX system featuring the silicon drift detector Ultim Max (Oxford Instruments, Abingdon, UK) and AztecLive as EDX software (version 4.4, Abingdon, UK). The sample surface was examined at a magnification ranging from 120× to 3000×. Backscattered and secondary electron images were obtained at an acceleration voltage of 10 kV and a probe current of 10.

## 5. Conclusions

To summarise, the evidence gathered in this study indicates that CSF pellets contain several identifiable brain-derived components, including wasteosomes, psammoma bodies, and deposits of disease-related protein aggregates, as well as other components like membranous remnants, fibrillar structures, and vesicles, the origin of which needs to be confirmed. The presence of such a variety of products and components has gone unnoticed until now and, therefore, has not been previously studied. These observations suggest that a detailed investigation of CSF pellets is warranted to determine whether they contain substances useful in the context of diagnosis or prognosis. Such studies need to demonstrate either (a) that sediment-derived measurements offer superior diagnostic accuracy than supernatant, (b) that pellet-based analysis detects biomarkers not accessible in the supernatant, or (c) that some potential novel markers are uniquely present in the pellet fraction. Consequently, future biomarker research should consider analysing these sediments or adapting existing methodologies to study uncentrifuged CSF directly. Investigating CSF pellets or uncentrifuged CSF may offer valuable insights into the pathophysiology of neurodegenerative diseases, ultimately improving early diagnosis and disease monitoring, reinforcing the translational link between basic research and therapeutic applications.

## Data Availability

The original contributions presented in this study are included in the article. Further inquiries can be directed to the corresponding authors.

## Abbreviations

The following abbreviations are used in this manuscript:

Aβ	Amyloid-β
Aβ40	Amyloid-β 40 isoform
Aβ42	Amyloid-β 42 isoform
AD	Alzheimer’s disease
AF	Alexa fluor
BB	Blocking buffer
CA	Corpora amylacea
Ca	Calcium
CSF	Cerebrospinal fluid
EDX	Energy-dispersive X-ray spectroscopy
FTLD	Frontotemporal lobar degeneration
FTLD-FUS	Frontotemporal lobar degeneration with FUS inclusions
FTLD-TDP	Frontotemporal lobar degeneration with TDP-43 inclusions
FTLD-tau	Frontotemporal lobar degeneration with tau pathology
FUS	Fused in sarcoma protein
GS	Glycogen synthase
H	Hydrogen
IgG	Immunoglobulin G
IgM	Immunoglobulin M
NFT	Neurofibrillary tangle
O	Oxygen
P	Phosphorus
p62	Ubiquitin-binding protein p62
PAS	Periodic acid–Schiff
PB	Phosphate buffer
PBS	Phosphate-buffered saline
PSP	Progressive supranuclear palsy
ROI	Region of interest
SEM	Scanning electron microscopy
SEM-EDX	Scanning electron microscopy with energy-dispersive X-ray spectroscopy
Si	Silicon
Simoa	Single-molecule array
TEM	Transmission electron microscopy
TDP-43	Transactive response DNA-binding protein 43
Ubi	Ubiquitin

## References

[1] Wilson D.M., Cookson M.R., Van Den Bosch L., Zetterberg H., Holtzman D.M., Dewachter I. (2023). Hallmarks of neurodegenerative diseases. Cell.

[2] Knopman D.S., Amieva H., Petersen R.C., Chételat G., Holtzman D.M., Hyman B.T., Nixon R.A., Jones D.T. (2021). Alzheimer disease. Nat. Rev. Dis. Primers.

[3] Spillantini M.G., Schmidt M.L., Lee V.M., Trojanowski J.Q., Jakes R., Goedert M. (1997). Alpha-synuclein in Lewy bodies. Nature.

[4] Grossman M., Seeley W.W., Boxer A.L., Hillis A.E., Knopman D.S., Ljubenov P.A., Miller B., Piguet O., Rademakers R., Whitwell J.L. (2023). Frontotemporal lobar degeneration. Nat. Rev. Dis. Primers.

[5] Mackenzie I.R.A., Neumann M., Bigio E.H., Cairns N.J., Alafuzoff I., Kril J., Kovacs G.G., Ghetti B., Halliday G., Holm I.E. (2010). Nomenclature and nosology for neuropathologic subtypes of frontotemporal lobar degeneration: An update. Acta Neuropathol..

[6] Blennow K., Hampel H., Weiner M., Zetterberg H. (2010). Cerebrospinal fluid and plasma biomarkers in Alzheimer disease. Nat. Rev. Neurol..

[7] Hansson O. (2021). Biomarkers for neurodegenerative diseases. Nat. Med..

[8] Molinuevo J.L., Blennow K., Dubois B., Engelborghs S., Lewczuk P., Perret-Liaudet A., Teunissen C., Parnetti L. (2014). The clinical use of cerebrospinal fluid biomarker testing for Alzheimer’s disease diagnosis: A consensus paper from the Alzheimer’s Biomarkers Standardization Initiative. Alzheimer’s Dement..

[9] Zetterberg H., Blennow K. (2021). Moving fluid biomarkers for Alzheimer’s disease from research tools to routine clinical diagnostics. Mol. Neurodegener..

[10] Alcolea D., Delaby C., Muñoz L., Torres S., Estellés T., Zhu N., Barroeta I., Carmona-Iragui M., Illán-Gala I., Santos-Santos M.Á (2021). Use of plasma biomarkers for AT(N) classification of neurodegenerative dementias. J. Neurol. Neurosurg. Psychiatry.

[11] Scialò C., Tran T.H., Salzano G., Novi G., Caponnetto C., Chiò A., Calvo A., Canosa A., Moda F., Caroppo P. (2020). TDP-43 real-time quaking induced conversion reaction optimization and detection of seeding activity in CSF of amyotrophic lateral sclerosis and frontotemporal dementia patients. Brain Commun..

[12] Smith R., Hovren H., Bowser R., Bakkar N., Garruto R., Ludolph A., Ravits J., Gaertner L., Murphy D., Lebovitz R. (2024). Misfolded alpha-synuclein in amyotrophic lateral sclerosis: Implications for diagnosis and treatment. Eur. J. Neurol..

[13] Teunissen C.E., Kimble L., Bayoumy S., Bolsewig K., Burtscher F., Coppens S., Das S., Gogishvili D., Fernandes Gomes B., Gómez de San José N. (2023). Methods to discover and validate biofluid-based biomarkers in neurodegenerative dementias. Mol. Cell. Proteom..

[14] Teunissen C.E., Petzold A., Bennett J.L., Berven F.S., Brundin L., Comabella M., Franciotta D., Frederiksen J.L., Fleming J.O., Furlan R. (2009). A consensus protocol for the standardization of cerebrospinal fluid collection and biobanking. Neurology.

[15] Ashton N.J., Brum W.S., Di Molfetta G., Benedet A.L., Arslan B., Jonatis E., Langhough R.E., Cody K., Wilson R., Carlsson C.M. (2023). Diagnostic accuracy of the plasma ALZpath pTau217 immunoassay to identify Alzheimer’s disease pathology. medRxiv.

[16] del Campo M., Mollenhauer B., Bertolotto A., Engelborghs S., Hampel H., Simonsen A.H., Kapaki E., Kruse N., Le Bastard N., Lehmann S. (2012). Recommendations to standardize preanalytical confounding factors in Alzheimer’s and Parkinson’s disease cerebrospinal fluid biomarkers: An update. Biomark. Med..

[17] Riba M., Augé E., Campo-Sabariz J., Moral-Anter D., Molina-Porcel L., Ximelis T., Ferrer R., Martín-Venegas R., Pelegrí C., Vilaplana J. (2019). Corpora amylacea act as containers that remove waste products from the brain. Proc. Natl. Acad. Sci. USA.

[18] Augé E., Cabezón I., Pelegrí C., Vilaplana J. (2017). New perspectives on corpora amylacea in the human brain. Sci. Rep..

[19] Sbarbati A., Carner M., Colletti V., Osculati F. (1996). Extrusion of corpora amylacea from the marginal glia at the vestibular root entry zone. J. Neuropathol. Exp. Neurol..

[20] Augé E., Duran J., Guinovart J.J., Pelegrí C., Vilaplana J. (2018). Exploring the elusive composition of corpora amylacea of human brain. Sci. Rep..

[21] Townsend L.E., Gilroy J., LeWitt P., Wolfe D.E., Pomara N., Weintraub J., Reitz D. (1987). Comparison of methods for analysis of CSF proteins in patients with Alzheimer’s disease. Neurochem. Pathol..

[22] Fagan A.M., Mintun M.A., Mach R.H., Lee S.Y., Dence C.S., Shah A.R., LaRossa G.N., Spinner M.L., Klunk W.E., Mathis C.A. (2006). Inverse relation between in vivo amyloid imaging load and cerebrospinal fluid Abeta42 in humans. Ann. Neurol..

[23] Pitschke M., Prior R., Haupt M., Riesner D. (1998). Detection of single amyloid beta-protein aggregates in the cerebrospinal fluid of Alzheimer’s patients by fluorescence correlation spectroscopy. Nat. Med..

[24] Nirmalraj P.N., Schneider T., Lüder L., Felbecker A. (2023). Protein fibril length in cerebrospinal fluid is increased in Alzheimer’s disease. Commun. Biol..

[25] Da Mesquita S., Fu Z., Kipnis J. (2018). The meningeal lymphatic system: A new player in neurophysiology. Neuron.

[26] Da Mesquita S., Louveau A., Vaccari A., Smirnov I., Cornelison R.C., Kingsmore K.M., Contarino C., Onengut-Gumuscu S., Farber E., Raper D. (2018). Functional aspects of meningeal lymphatics in ageing and Alzheimer’s disease. Nature.

[27] Jiang-Xie L.F., Drieu A., Kipnis J. (2024). Waste clearance shapes aging brain health. Neuron.

[28] Salvador A.F.M., Abduljawad N., Kipnis J. (2024). Meningeal lymphatics in central nervous system diseases. Annu. Rev. Neurosci..

[29] Al-Diwani A., Provine N.M., Murchison A., Laban R., Swann O.J., Koychev I., Sheerin F., Da Mesquita S., Heslegrave A., Zetterberg H. (2025). Neurodegenerative fluid biomarkers are enriched in human cervical lymph nodes. Brain.

[30] Riba M., Del Valle J., Augé E., Vilaplana J., Pelegrí C. (2021). From corpora amylacea to wasteosomes: History and perspectives. Ageing Res. Rev..

[31] Alsina R., Riba M., Pérez-Millan A., Borrego-Écija S., Aldecoa I., Romera C., Balasa M., Antonell A., Lladó A., Compta Y. (2024). Increase in wasteosomes (corpora amylacea) in frontotemporal lobar degeneration with specific detection of tau, TDP-43 and FUS pathology. Acta Neuropathol. Commun..

[32] Averback P. (1981). Parasynaptic corpora amylacea in the striatum. Arch. Pathol. Lab. Med..

[33] Busard H.L., Span J.P., Renkawek K., Renier W.O., Gabreëls F.J., Slooff J.L., Van’t Hof M.A. (1994). Polyglucosan bodies in brain tissue: A systematic study. Clin. Neuropathol..

[34] Cavanagh J.B. (1999). Corpora-amylacea and the family of polyglucosan diseases. Brain Res. Rev..

[35] Cissé S., Perry G., Lacoste-Royal G., Cabana T., Gauvreau D. (1993). Immunochemical identification of ubiquitin and heat-shock proteins in corpora amylacea from normal aged and Alzheimer’s disease brains. Acta Neuropathol..

[36] Riba M., Del Valle J., Romera C., Alsina R., Molina-Porcel L., Pelegrí C., Vilaplana J. (2023). Uncovering tau in wasteosomes (corpora amylacea) of Alzheimer’s disease patients. Front. Aging Neurosci..

[37] Singhrao S.K., Morgan B.P., Neal J.W., Newman G.R. (1995). A functional role for corpora amylacea based on evidence from complement studies. Neurodegeneration.

[38] Wilhelmus M.M., Verhaar R., Bol J.G., van Dam A.M., Hoozemans J.J., Rozemuller A.J., Drukarch B. (2011). Novel role of transglutaminase 1 in corpora amylacea formation?. Neurobiol. Aging.

[39] Wander C.M., Tseng J.H., Song S., Al Housseiny H.A., Tart D.S., Ajit A., Shih Y.-Y.I., Lobrovich R., Song J., Meeker R.B. (2020). The accumulation of tau-immunoreactive hippocampal granules and corpora amylacea implicates reactive glia in tau pathogenesis during aging. iScience.

[40] Riba M., Del Valle J., Molina-Porcel L., Pelegrí C., Vilaplana J. (2022). Wasteosomes (corpora amylacea) as a hallmark of chronic glymphatic insufficiency. Proc. Natl. Acad. Sci. USA.

[41] Riba M., Augé E., Tena I., Del Valle J., Molina-Porcel L., Ximelis T., Vilaplana J., Pelegrí C. (2021). Corpora amylacea in the human brain exhibit neoepitopes of a carbohydrate nature. Front. Immunol..

[42] Riba M., Campo-Sabariz J., Tena I., Molina-Porcel L., Ximelis T., Calvo M., Ferrer R., Martín-Venegas R., del Valle J., Vilaplana J. (2022). Wasteosomes (corpora amylacea) of human brain can be phagocytosed and digested by macrophages. Cell Biosci..

[43] Riba M., Romera C., Alsina R., Alsina-Scheer G., Pelegrí C., Vilaplana J., del Valle J. (2023). Analyzing the Virchow pioneering report on brain corpora amylacea: Shedding light on recurrent controversies. Brain Struct. Funct..

[44] Virchow R. (1854). Ueber eine im Gehirn und Rückenmark des Menschen aufgefundene Substanz mit der chemischen Reaction der Cellulose. Arch. Pathol. Anat. Physiol. Klin. Med..

[45] Jovanović I., Ugrenović S., Antić S., Stefanović N., Mihailović D. (2007). Morphometric and some immunohistochemical characteristics of human choroid plexus stroma and psammoma bodies. Microsc. Res. Tech..

[46] Jovanović I., Ugrenović S., Vasović L., Petrović D., Cekić S. (2010). Psammoma bodies—Friends or foes of the aging choroid plexus. Med. Hypotheses.

[47] Živković V.S., Stanojković M.M., Antić M.M. (2017). Psammoma bodies as signs of choroid plexus ageing—A morphometric analysis. Vojnosanit. Pregl..

[48] Virchow R. (1865). Die Krankhaften Geschwülste.

[49] Montine T.J., Phelps C.H., Beach T.G., Bigio E.H., Cairns N.J., Dickson D.W., Duyckaerts C., Frosch M.P., Masliah E., Mirra S.S. (2012). National Institute on Aging-Alzheimer’s Association guidelines for the neuropathologic assessment of Alzheimer’s disease: A practical approach. Acta Neuropathol..

[50] Schneider C.A., Rasband W.S., Eliceiri K.W. (2012). NIH Image to ImageJ: 25 years of image analysis. Nat. Methods.

